# Adaptive Evolution of *Aurantiochytrium limacinum* for Efficient Production of Docosahexaenoic Acid Under Acidic and High-Temperature Conditions

**DOI:** 10.3390/microorganisms13092022

**Published:** 2025-08-29

**Authors:** Tanapawarin Rampai, Rujirek Nopgasorn, Kobkul Laoteng, Siwaporn Wannawilai

**Affiliations:** Industrial Bioprocess Technology Research Team, Functional Ingredients and Food Innovation Research Group, National Center for Genetic Engineering and Biotechnology (BIOTEC), National Science and Technology Development Agency (NSTDA), Thailand Science Park, Phahonyothin Road, Khlong Nueng, Khlong Luang, Pathum Thani 12120, Thailand; tanapawarin.ram@biotec.or.th (T.R.); rujirek.nop@biotec.or.th (R.N.); kobkul@biotec.or.th (K.L.)

**Keywords:** *Aurantiochytrium limacinum*, acid tolerance, adaptive laboratory evolution, docosahexaenoic acid, high temperature, kinetic model

## Abstract

Acid- and heat-tolerant industrial microbial strains are crucial for biotechnological production because they minimize the risk of microbial contamination and reduce energy consumption associated with cooling requirements. Here, adaptive laboratory evolution (ALE) of *Aurantiochytrium limacinum* was performed to improve the capability of the strain to produce docosahexaenoic acid (DHA) under acidic and high-temperature conditions. A stepwise increase from 30 to 38 °C was applied during cultivation at pH 4.5. After 30 cycles of high-temperature exposure (34 °C), an adaptive strain (BBF002) was obtained. Cell growth and DHA production of BBF002 were higher than those of the parental strain (BBF001) by 32.95 and 7.12%, respectively, at pH 4.5 and 30 °C. Based on the experimental data obtained using glucose as a carbon source, a kinetic model was developed to describe cell growth, biomass maintenance, and DHA, and we used other metabolite methods to produce the native, parental, and adaptive strains. The growth traits of the three strains could be adequately described through logistic modeling. DHA was found to be a mixed-growth product produced during exponential and stationary phases, according to the Luedeking–Piret equation.

## 1. Introduction

Docosahexaenoic acid (DHA; C22:6 n-3) is a long-chain polyunsaturated fatty acid (LC-PUFA) in the n-3 series, which plays vital roles in the brain and eye development in infants. Moreover, it is effective in the prevention and treatment of chronic diseases, such as hypertension, atherosclerosis, type-2 diabetes mellitus, depression, and some cancers [1,2,3]. Thus, DHA has been widely used as a dietary supplement and functional ingredient in food and feed products. This LC-PUFA is commercially produced from marine sources, such as fish and algae [4,5,6]. However, concerns regarding heavy metal and toxic compound contamination in marine environments and overfishing have led to the development of alternative sources for sustainable DHA production [7,8]. Considerable attention has been paid to the development of microbial DHA production methods. Among the DHA-producing strains, the thraustochytrids, *Aurantiochytrium* spp., have the potential to produce substantial amounts of DHA-rich oils [9,10,11]. DHA derived from *Aurantiochytrium* has been proven to be safe for human consumption. The DHA-producing strains require specific culture conditions to promote cell growth and lipid production yield, such as a pH range of 6–7 and a temperature of 28 °C [12,13,14]. However, neutral pH conditions can lead to cross-contamination with unwanted microbes, and operating at low temperatures increases energy consumption, thereby increasing production costs.

Strain improvement can be used to overcome the challenges in industrial production processes and is implemented either via metabolic engineering or adaptive laboratory evolution (ALE) approach. ALE is a powerful strategy for phenotypic optimization through genotypic changes induced by prolonged cultivation under selective pressure, including nutritional and environmental stress [15,16], such as low temperature [17,18,19], high osmotic pressure [20], high salt stress [21,22], high oxygen levels [23,24], and high temperatures [25]. Recently, an acid-tolerant adaptive *A. limacinum* strain BBF001 was generated via ALE and was shown to possess the ability to grow under acidic conditions (pH 4.0–5.5). Notably, the acid-tolerant strain maintained normal growth and DHA content, similar to those of the native strain (*A. limacinum* TBRC-BCC55172). This strain offers scope for further improvement by incorporating high-temperature tolerance.

In this study, to improve the tolerance of the strain BBF001 to high temperatures, we performed ALE under high-temperature conditions. Kinetic models for DHA production were generated using experimental data on the growth of all strains, particularly at the DHA-accumulating stages, to describe the correlation between growth and DHA production. This study demonstrated a stepwise ALE approach to generate an *Auranthiochytrium* strain with acid and high-temperature tolerance that exhibited good performance for DHA production via submerged fermentation.

## 2. Materials and Methods

### 2.1. Microbial Strain and Inoculum Preparation

All *A. limacinum* strains—native (TBRC-BCC55172), acid-tolerant (parental strain BBF001), and acid- and temperature-tolerant (adaptive strain BBF002)—were preserved in 20% (*v*/*v*) glycerol at −80 °C.

For inoculum preparation, *A. limacinum* was cultured in the production medium, which consisted of 40 g/L glucose, 10 g/L yeast extract, 10 g/L peptone, 0.4 g/L MgSO_4_.7H_2_O, 0.02 g/L MnSO_4_.5H_2_O, 0.02 g/L FeSO4.7H_2_O, and 30 g/L NaCl. The pH of the medium was adjusted to 4.5. The cultivation was performed in a 250 mL Erlenmeyer flask containing 50 mL medium at 25 °C by agitating at 200 rpm for 72 h.

### 2.2. Adaptive Laboratory Evolution

The adaptive evolution of the parental strain (BBF001) was performed in 250 mL Erlenmeyer flasks using a long-term serial transfer procedure. Subculturing of the cells in liquid medium was performed using a stepwise increase in temperature from 30 to 38 °C. The culture was performed at a shaking rate of 200 rpm. The pH of the medium was adjusted to 4.5. The culture (10%, *v*/*v*) was transferred to a fresh medium every 3 days. The temperature was controlled by increasing 2 °C for every five subcultures of fermentation (one round). The adaptive strains capable of growing at each temperature (ALE-T30, ALE-T32, ALE-T34, and ALE-T36) were picked and preserved in 20% (*v*/*v*) glycerol solution at −80 °C. All adaptive strains were cultured in shake flasks to investigate their cell growth and DHA production.

The ALE-T34 strain of *A. limacinum* was selected to maintain growth under acidic and high-temperature conditions. The adaptive evolution was further carried out at a controlled temperature of 34 °C with shaking at 200 rpm for 30 cycles. The culture (10%, *v*/*v*) was transferred to a fresh medium every three days—this was defined as one cycle. After 30 cycles, the endpoint adaptive strain of *A. limacinum* (BBF002) was obtained, which was preserved in 20% (*v*/*v*) glycerol at −80 °C for further study.

### 2.3. Submerged Fermentation

The growth performance and DHA production of the adaptive strains were evaluated using shake-flask cultivation at various temperatures (25, 30, and 35 °C) compared with those of native and parental strains. The cultures were grown in the production medium with an initial pH of 4.5 using a 10% (*v*/*v*) inoculum and a shaking rate of 200 rpm for 5 days. All experiments were performed independently in triplicate.

### 2.4. Analytical Methods

The fermented cultures were centrifuged to harvest the cells. The cells were dried at 60 °C in a hot-air oven until a constant weight was achieved. The cell biomass was measured as dry cell weight (DCW). The supernatant was used to measure the pH and residual glucose concentration. The sugar concentration in the filtered broth was analyzed using a high-performance liquid chromatography system (HPLC; Ultimate 3000, Thermo, Waltham, MA, USA), equipped with a refractive index detector and an Aminex^®®^ HPX-87H ion exclusion column (Bio-Rad Laboratories, Hercules, CA, USA). The chromatography was performed in an isocratic mode at 60 °C for 30 min using 18 mM H_2_SO_4_ as the mobile phase at a flow rate of 0.6 mL/min. The residual glucose in the fermented broths was quantified using a calibration curve of a glucose standard generated for a concentration range of 0.1–10.0 mg/mL.

For fatty acid analysis, fatty acid methyl esters (FAMEs) were prepared from dried cells using a method modified from that described by Lepage and Roy [26]. The samples were analyzed using a gas chromatography (GC) system (Agilent 7890B, Santa Clara, CA, USA), equipped with a flame ionization detector and an HP-88 capillary column (100 m × 250 µm × 0.2 µm, Agilent, USA). Individual fatty acids were identified based on their retention times compared with the respective FAME standards (Sigma, Ronkonkoma, NY, USA) and quantified using nonadecanoic acid (C19:0) as an internal fatty acid standard.

### 2.5. Data Analysis

All experimental data are expressed as mean values with standard deviation (SD). Duncan’s one-way ANOVA test (SPSS 11.5 software for Windows; SPSS Inc., Chicago, IL, USA) was used for the statistical analysis and calculation of kinetic fermentation parameters. Heatmap plot analysis of fatty acid composition (%, *w*/*w*) was performed using TBtools software (https://github.com/CJ-Chen/TBtools/releases, accessed on 22 August 2025). The kinetic parameters of fermentation were calculated from time zero (*t*_1_; day) to the point (*t*_2_; day) at which the DHA concentration (*C*_P_; g/L) was the maximum (5 days of fermentation time), to obtain comparable results among a set of experiments.

For determining the rates of operation, the rates of biomass production (*Q*_X_; g/L d), glucose consumption (*Q*s; g/L d), and DHA production (*Q*_P_; g/L d) were calculated as follows:

(1) $$Q_{X}=\;\frac{C_{X,2}-C_{X,1}}{t_{2}-t_{1}}$$

(2) $$Q_{S}=-\frac{C_{S,2}-C_{S,1}}{t_{2}-t_{1}}$$

(3) $$Q_{P}=\frac{C_{P,2}-C_{P,1}}{t_{2}-t_{1}}$$

where *C*_X_ is biomass concentration or DCW (g/L), *C*_S_ is glucose concentration (g/L), and *C*_P_ is DHA concentration (g/L).

The specific growth rate (µ; d^−1^) was calculated by dividing the biomass production rate by the average biomass concentration, as follows:

(4) $$\mu \;=\;\frac{1}{\left(\frac{C_{X,1}\;+\;C_{X,2}}{2}\right)}Q_{X}$$

For the yield coefficients, the biomass yield (*Y*_X/S_; g/g) was calculated from the amount of whole biomass per unit of consumed glucose, and the DHA yield (*Y*_P/S_; g/g) was calculated from the amount of DHA produced per unit of consumed glucose as follows:

(5) $$Y_{X/S}\;=\;\frac{C_{X,2}-C_{X,1}}{C_{S,1}-C_{S,2}}$$

(6) $$Y_{P/S}=\frac{C_{P,2}-C_{P,1}}{C_{S,1}-C_{S,2}}$$

### 2.6. Kinetic Modeling of Growth and DHA Production

The kinetic modeling was based on the oleaginous features of the native strain and evolved strains BBF001 and BBF002 of *A. limacinum*. The growth profiles (*dC*_X_/*dt*) described the biomass concentration at any time depending on the specific growth rate. The growth was characterized to follow a logistic equation in which the specific growth rate is related to the cell-carrying capacity [27], as follows:

(7) $$\frac{dC_{X}}{\mathrm{dt}}\;=\;µC_{X}$$

(8) $$\mu \;=\mu_{\max}\left(1-\frac{C_{X}}{C_{\mathrm{Xm}}}\right)$$

where µ is specific growth rate (d^−1^), µ_max_ is the maximum specific growth rate (d^−1^), and *C*_Xm_ is the maximum biomass concentration (g/L).

The consumption rate of a substrate (*dC*_S_/*dt*) depends on the microbial growth rate, which includes lipids and other intracellular metabolite products and biomass maintenance, in a way that can be expressed using the following equation:

(9) $$\frac{dC_{S}}{\mathrm{dt}}\;=\;-\left[\left(\frac{1}{Y_{X/S}}\right)\left(\frac{dC_{X}}{\mathrm{dt}}\right)\;+\;m_{S}C_{X}\right]$$

where *m*_S_ is the biomass maintenance coefficient (g/g d) and *Y*_X/S_ is the biomass yield on the substrate (g/g). The value of *Y*_X/S_ was 0.45 g/g, which was estimated from the calculation based on the biomass molecular formula (CH_1.79_ O_0.5_ N_0.2_) and glucose as the substrate [28].

The DHA production rate (*dC*_P_/*dt*) depends on the biomass concentration according to the Luedeking–Piret equation as follows:

(10) $$\frac{dC_{P}}{\mathrm{dt}}\;=\;(\alpha µ\;+\;\beta )C_{X}$$

where α is a growth-associated product formation coefficient (g/g), and β is a non-growth-associated product formation coefficient (g/g d).

The model parameters, µ_max_, *C*_Xm_, *m*_S_, α, and β, were estimated using the Berkeley Madonna 10 software for Windows (http://www.berkeleymadonna.com/, accessed on 18 July 2025) from the best fit of kinetic models to the experimental data. The parameter values with a determination coefficient (*R*^2^) approximately 1.0 were considered as the best fit between the model-predicted and the experimental values [29], as defined below.

(11) $$R^{2}=\frac{{\sum \left(C_{\mathrm{cal}}-C_{\exp,\mathrm{ave}}\right)}^{2}}{{\sum \left(C_{\mathrm{cal}}-C_{\exp,\mathrm{ave}}\right)}^{2}+\sum {\left(C_{\mathrm{cal}}-C_{\exp}\right)}^{2}}$$

where *C*_cal_ is the value calculated from the model, *C*_exp_ is the experimental value, and *C*_exp,ave_ is the average of all experimental data derived from the variables studied.

## 3. Results and Discussion

### 3.1. Response of the Adaptive Strains to Normal Culture Conditions

During ALE of acid-tolerant *A. limacinum* (parental strain) using a stepwise temperature approach, the cells grew well over a temperature range of 30–36 °C. These strains were designated ALE-T30, ALE-T32, ALE-T34, and ALE-T36. However, the ALE-T36 strain showed poor growth when further subcultured at 38 °C. Thus, we investigated the fermentation characteristics of the four adapted strains (ALE-T30, ALE-T32, ALE-T34, and ALE-T36) and compared them with those of the parental strain (BBF001).

**Figure 1:**
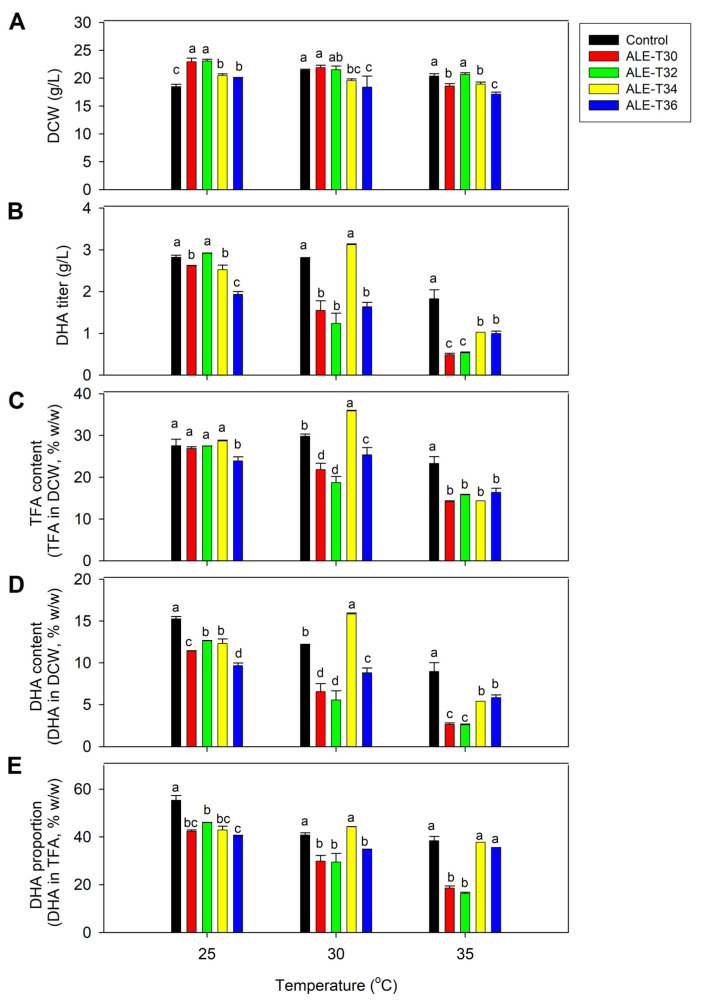
Comparative analysis of biomass and lipid production between the acid-tolerant (BBF001) and four adaptive laboratory evolution (ALE) strains grown at 25, 30 and 35 °C for 5 days. (**A**) Dry cell weight (g/L); (**B**) docosahexaenoic acid (DHA) titer (g/L); (**C**) TFA content (TFA in DCW, % *w*/*w*); (**D**) DHA content (DHA in DCW, % *w*/*w*); and (**E**) DHA proportion (DHA in TFA, % *w*/*w*). Different lowercase letters represent significant differences between the same temperature test groups (*p* < 0.05).

The cell growth of ALE strains was primarily investigated at 25 °C, which is the optimal temperature for *Auranthiochytrium* spp. The results showed that the biomass (DCW) titers of the four adaptive strains grown for 5 days were higher than those of the BBF001 strain used as a control (Figure 1A). These results indicated that the ALE strains had superior growth performance compared with the parental strain at this temperature. The maximum cell dry weight of 23.10 g/L was obtained for ALE-T32, which was 24.9% higher than that of the BBF001 strain. Notably, no difference in DHA titers (Figure 1B) was noted between the ALE-T32 and BBF001 strains (2.82–2.92 g/L). Moreover, the total fatty acid (TFA) content of the ALE strains was comparable to that of the parental strain (Figure 1C), except for ALE-T36. However, the DHA content (Figure 1D) and proportion (Figure 1E) in all adaptive strains were lower than those in the control, indicative of a trade-off phenotype resulting from adaptive evolution.

### 3.2. Response of the ALE Strains to High-Temperature Cultivation

To characterize the growth and DHA phenotypes of ALE strains in response to high temperatures, they were grown at 30 and 35 °C for 5 days (Figure 1). As expected, the ALE strains were able to grow and simultaneously accumulate DHA. At 30 °C, the growth of ALE-T30 and ALE-T32 was comparable to that of the parental strain at 22.30–23.70 g/L (Figure 1A). However, ALE-T34 was the only strain to exhibit DHA titer and proportion comparable to the parental strain. Moreover, its TFA and DHA contents were 20.29% and 30.31% higher than that of the control (Figure 1B,E), although its biomass (19.70 g/L) was lower than that of the parental strain (Figure 1A). When the culture temperature increased to 35 °C, all the adaptive and parental strains showed lower biomass than the culture at 25 and 30 °C (Figure 1A). The maximum cell dry weight of 20.70 g/L was obtained from ALE-T32, which was not significantly different from that of the control (Figure 1A). For TFA and DHA production, although all the adaptive strains showed low TFA accumulation and DHA production (Figure 1B,D), the DHA proportion (~38% of TFA) in ALE-T34 remained relatively high, comparable to that of the control (Figure 1E).

Based on these results, the ALE-T34 strain was selected for further adaptive evolution at a controlled temperature of 34 °C for 30 cycles to achieve improved performance with a stable phenotypic trait, and it was named *A. limacinum* strain BBF002.

### 3.3. Growth of Native and Adaptive Strains of A. limacinum

**Figure 2:**
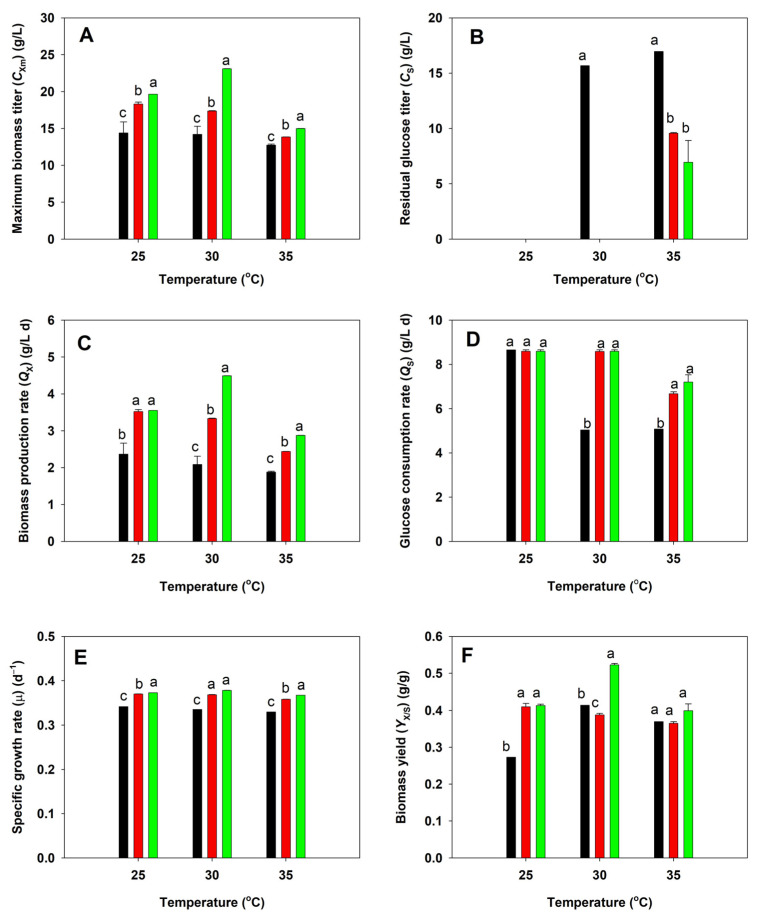
Comparative analysis of growth between the native (black), acid-tolerant (BBF001; red), and acid- and temperature-tolerant (BBF002; green) strains grown at different temperatures for 5 days. (**A**) Maximum biomass titer; (**B**) residual glucose titer; (**C**) biomass production rate; (**D**) glucose consumption rate; (**E**) specific growth rate; and (**F**) biomass yield from substrate. Different lowercase letters represent significant differences between the same temperature test groups (*p* < 0.05).

The native, acid-tolerant (BBF001), and acid- and high-temperature-tolerant (BBF002) strains of *A. limacinum* were cultured in shake flasks using glucose as a substrate at pH 4.5 at 25, 30, and 35 °C. The cell growth and glucose consumption of all strains were measured and compared. After 5 days of fermentation, the maximum biomass titer of the native strain was significantly lower than that of the BBF001 and BBF002 strains at all temperatures (Figure 2A), and its growth decreased as the culture temperature increased. The result corresponded to its glucose consumption. The native strain consumed all the glucose within 5 days of fermentation when it was cultured at 25 °C, and its consumption decreased as the culture temperature increased. Therefore, the residual glucose increased following the increase in culture temperature (Figure 2B). These behaviors of the native strain were influenced by low pH and high-temperature conditions, which were unsuitable for cell propagation. Similarly to that for the native strain, the maximum biomass titer of BBF001 was high (18.32 g/L) at 25 °C, which is the optimal temperature for *Auranthiochytrium* spp., and the decreased biomass titer was obtained at higher culture temperatures (Figure 2A). Although BBF001 could exhaust glucose when cultured at 30 °C (Figure 2B), the lower biomass titer was obtained because some glucose was used for cell maintenance from the effect of adaptive evolution [30]. In contrast, the BBF002 strain, which had evolved under acidic and high-temperature conditions, grew well at higher temperature (30 °C) yielding the highest maximum biomass titer (23.10 g/L) and could consume all glucose within 5 days of fermentation as did the BBF001 strain (Figure 2B). However, because of adaptation under high temperatures, the growth of BBF002 was higher than that of native and BBF001 strains at high temperatures. The lowest maximum biomass titers were observed when all the strains were cultured at 35 °C (Figure 2A). From the growth kinetics parameters (Figure 2C,F), it was evident that cultivation of BBF002 at all temperatures resulted in high biomass production rate (*Q*_X_) (Figure 2C), glucose consumption rate (*Q*_S_) (Figure 2D), specific growth rate (µ) (Figure 2E), and biomass yield from substrate (*Y*_X/S_) (Figure 2F). In particular, cultivation at 30 °C resulted in the highest *Q*_X_ (4.494 g/L d), *Q*_S_ (8.595 g/L d), µ (0.379 d^−1^), and *Y*_X/S_ (0.523 g/g).

### 3.4. DHA Production of Native and Adaptive Strains of A. limacinum

**Figure 3:**
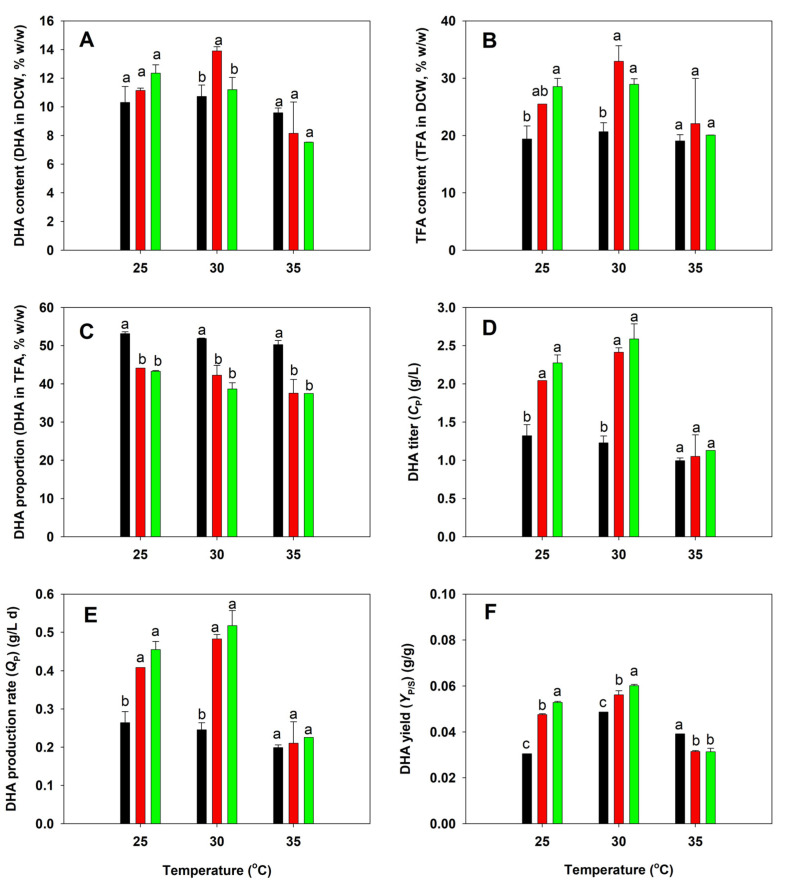
Comparative analysis of docosahexaenoic acid (DHA) production between the native (black), acid-tolerant (BBF001; red), and acid- and temperature-tolerant (BBF002; green) strains grown at different temperatures for 5 days. (**A**) DHA content in dry cell weight (DCW); (**B**) total fatty acid (TFA) content in DCW; (**C**) DHA proportion in TFA; (**D**) DHA titer; (**E**) DHA production rate; and (**F**) DHA yield from the substrate. Different lowercase letters represent significant differences between the same temperature test groups (*p* < 0.05).

DHA production by the three *A. limacinum* strains was shown in Figure 3. The highest DHA content was found in the BBF001 strain grown at 30 °C (13.90% in DCW) (Figure 3A). At the same temperature, a high TFA content was detected in the BBF001 and BBF002 strains, which accounted for 28.94–32.97% of the DCW (Figure 3B). Although the BBF001 and BBF002 strains had a high DHA and TFA content, a lower DHA proportion (~40% of TFA) was found at all the temperatures tested (Figure 3C), which might be the result of the trade-off phenotypes of adaptive evolution. However, due to the high biomass titers of BBF001 and BBF002, the DHA titers and DHA production rates in both the cultures were high, especially at 30 °C (2.42–2.59 g/L and 0.48–0.52 g/L d, respectively) as shown in Figure 3D,E. When the DHA yield was considered together, BBF002 was the best strain for DHA production at 30 °C among the tested strains (Figure 3F).

**Figure 4:**
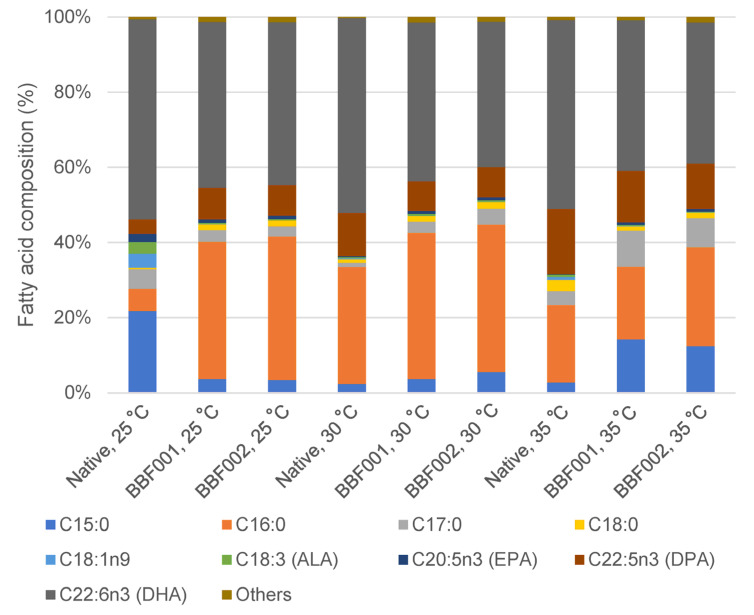
Fatty acid composition of the native, acid-tolerant strain (BBF001), and acid- and temperature-tolerant (BBF002) strains grown at different temperatures on day 5 of cultivation.

**Figure 5:**
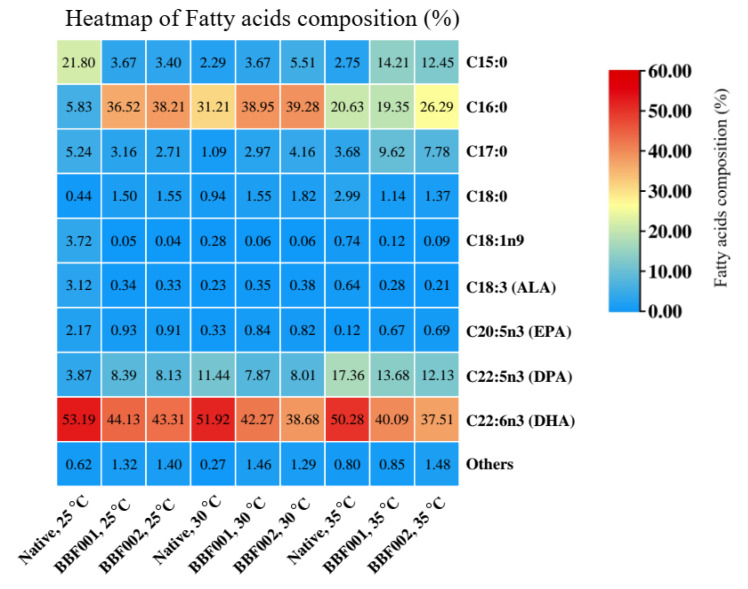
Heatmap analysis of fatty acid composition in the native and evolved strains (BBF001 and BBF002) under different cultivation temperatures (25, 30, and 35 °C).

The fatty acid profiles after adaptation and cultivation at different temperatures are shown in Figure 4. The fatty acid composition changed after adaptation. Under normal temperature, the fatty acids in the native strain mainly comprised 21.80% C15:0 and 53.19% DHA, whereas the BBF001 and BBF002 strains contained C16:0 and DHA as the major fatty acids. The percentage of C16:0 increased from 5.83% in the native strain to 36.52% and 38.21% in the BBF001 and BBF002 strains, respectively. Moreover, the docosapentaenoic acid (DPA) content in adaptive strains increased to 8.13–8.39% whereas it was 3.87% in the native strain. However, the composition of DHA decreased to 44.13% and 43.31% in the BBF001 and BBF002 strains, respectively. The increase in DPA content was similar to that in the adaptation of *Schizochytrium* sp. CCTCC M209059 under high temperatures [12]. However, changes in the fatty acid composition were also observed when cultivated at high temperatures. The percentage of C16:0 in the native and adaptive strains was significantly increased when the culture temperature increased to 30 °C and then decreased at 35 °C. Therefore, the highest C16:0 proportions in the native, BBF001, and BBF002 strains were 31.21, 38.95, and 39.28%, respectively, at 30 °C. Unlike C15:0, increased temperatures decreased the proportion of C15:0 in the native strain, but increased it in the adaptive strains. Therefore, the highest C15:0 content was found at 25 °C, being approximately 21.80% of that in the native strain, whereas it was 14.21 and 12.45% in the adaptive BBF001 and BBF002 strains at 35 °C. For polyunsaturated fatty acids, the increased culture temperature increased the proportion of DPA but decreased that of DHA in all the strains; the highest composition of DPA in the native, BBF001, and BBF002 strains was 17.36, 13.68, and 12.13%, respectively, at 35 °C and that of DHA was 53.19, 44.13, and 43.31% at 25 °C, respectively. Moreover, the change in fatty acid composition from heatmap analysis (Figure 5) emphasized significant differences between the native and BBF strains at various temperatures ranging from 25 to 35 °C. The native strain was characterized by higher levels of DHA coupled with lower amounts of C16:0. In contrast, the BBF strains, especially at temperatures between 25 and 30 °C, exhibited a higher concentration of C16:0 and a diminished presence of DHA compared to the native strain.

C16:0 was the most abundant saturated fatty acid (SFA) of all strains, with particularly high proportions detected in BBF001 and BBF002 at 25–30 °C (orange–red intensity). This indicated that the adaptive strains have a stronger tendency to accumulate saturated fatty acids compared with the native strain. In contrast, minor SFAs such as C15:0, C17:0, and C18:0 were obtained at very low proportions (predominantly blue). For polyunsaturated fatty acids (PUFAs), DHA was consistently the dominant PUFA under all strains and cultivation temperatures at 25–35 °C (intense red signal). DPA was detected at moderate proportion with slightly higher accumulation in adaptive strains compared to the native strain. Other PUFAs, eicosapentaenoic acid (EPA) and α-linolenic acid (ALA) were found only in small amounts (mostly blue). Moreover, it was found that the effect of temperature variation (25, 30, and 35 °C) had no influence on DHA proportions. However, temperature had a clear effect on C16:0 accumulation. Therefore, the strain improvement by ALE (BBF strains) appeared to have the metabolic flux toward enhanced synthesis of saturated fatty acids, while maintaining a high capacity for DHA biosynthesis.

From the results of this study, ALE based on a long-term serial transfer procedure under acidic conditions (pH 4.5) and high temperatures (34 °C) as the stress inducers resulted in the generation of the adaptive stain (BBF002), which could increase cell growth and DHA production after 30 cycles of adaptation. An acidic condition was used to improve the DHA content, whereas high temperatures was applied to stimulate cell growth. Acidic condition was found to be beneficial for DHA synthesis because it might promote the metabolic fluxes of the polyketide synthase pathway (PKS) pathway and activate the related key enzymes to enhance lipid accumulation and DHA synthesis during DHA production [31,32]. Temperature was an important factor that affected cell growth and fatty acid profiles [33,34,35,36]. At high temperatures, the content of long-chain fatty acids was high, whereas the proportion of polyunsaturated fatty acids (PUFAs) in total fatty acids was high at low temperature, correlating with the fluidity and rigidity of membrane [37]. In this study, high-temperature adaptation enhanced the heat resistance of BBF002, biomass titer, and cell growth but decreased the DHA content when compared with that in the acidic tolerant strain (BBF001). However, because of the highest maximum biomass titer (23.10 g/L) of BBF002, the highest DHA titer of 2.59 g/L was obtained at pH 4.5 and temperature 30 °C, which were 32.95% and 7.12% of the biomass and DHA titers of the parental strain (BBF001), respectively. Many other researchers have performed adaptive evolution based on a long-term serial transfer procedure using temperature or acidic conditions as stress inducers for improving DHA production. Sun et al. [18] reported the two-factor ALE strategy based on associated low temperature and high salinity to improve the DHA production capacity of *Schizochytrium* sp HX-308. Low temperature could improve the DHA content, and high salinity was useful to stimulate lipid accumulation and enhance the antioxidative defense systems in *Schizochytrium* sp. Hu et al. [25] performed ALE in *Schizochytrium* sp. CCTCC M209059 using high temperatures to enhance its thermotolerance in DHA production. After 70 cycles at high temperatures (34.5 °C), the adaptive strain exhibited a higher growth rate and lower temperature sensitivity and increased DHA concentration—4.33 times of that in the starting strain—at 34 °C. Moreover, Ding et al. [38] used multiple factor-based adaptive laboratory evolution strategy, combining high dissolved oxygen, low temperature, and citric acid-induced acidity conditions, to enhance DHA production in *Aurantiochytrium* sp. PKU#Mn16. This strategy yielded significant increases of 106.3% in biomass, 243.8% in total fatty acid yield, and 171.4% in DHA yield.

### 3.5. Kinetic Modeling of Growth and DHA Production

**Figure 6:**
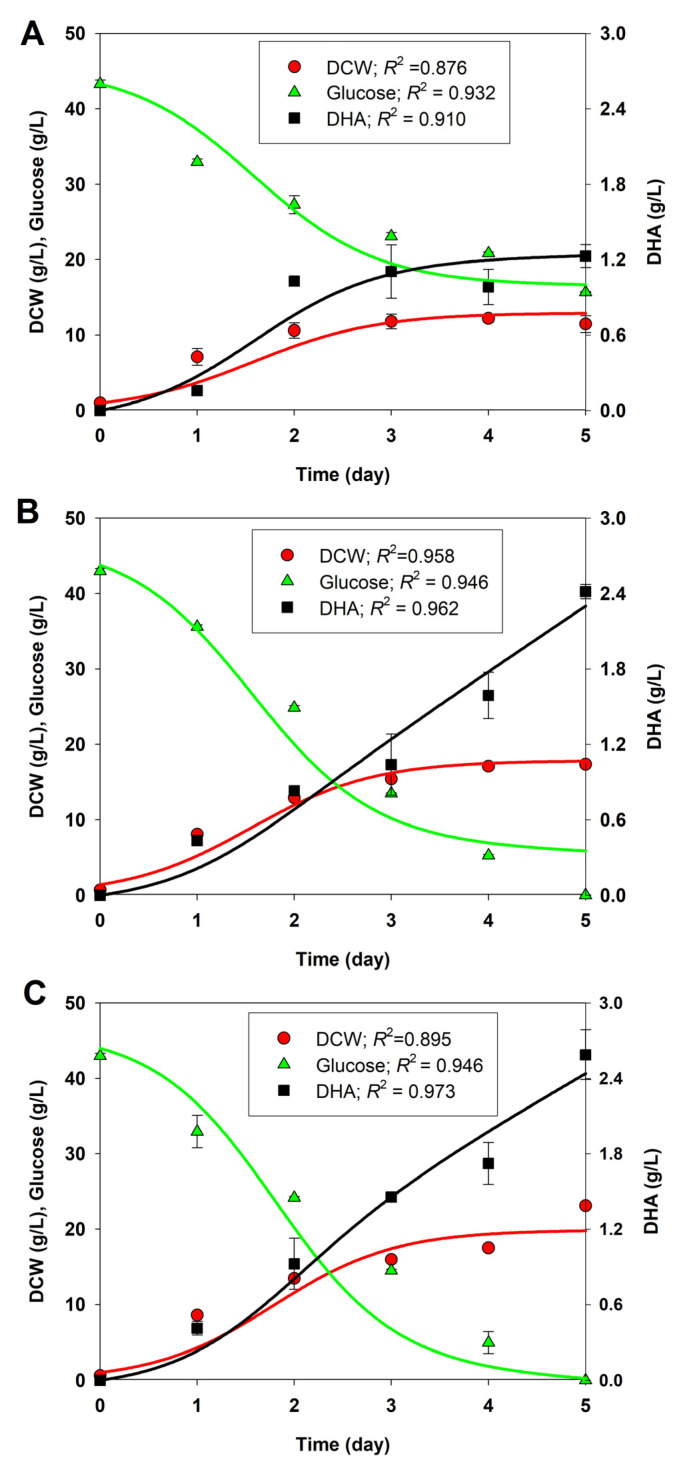
Comparison of the measured data (symbols) and the model-predicted profiles (lines) for the dry cell weight (DCW; circle), glucose (triangle), and DHA (square) titers in shake-flask cultures of the native (**A**), BBF001 (**B**), and BBF002 (**C**) strains at pH 4.5 and 30 °C.

Considering the improved performance of BBF002 with regard to biomass (Figure 2) and DHA production (Figure 3) at pH 4.5 and 30 °C, we chose the experimental data of growth, glucose consumption, and DHA production under these cultivation conditions for kinetic modeling. The results of this study were compared with those obtained for native and BBF001 strains cultured under the same conditions. The experimental data for cell growth, glucose consumption, and DHA production for the native, BBF001, and BBF002 strains cultivated at pH 4.5 and 30 °C were used to construct the kinetic models. The kinetics of cell growth, glucose consumption, and DHA production, described by Equations (7)–(10) (see Section 2), were used to simulate the models, along with the experimental data for the cultures. As shown in Figure 6, the growth, glucose consumption, and DHA production profiles for the native (Figure 6A), BBF001 (Figure 6B), and BBF002 (Figure 6C) strains fitted well with the experimental data (*R^2^* > 0.87), indicating that the growth traits of the three strains could be described well by a logistic model using glucose as the carbon substrate. Glucose was consumed to produce biomass, including lipids, DHA, and other intracellular metabolites, as well as for biomass maintenance. DHA could be produced as a mixed-growth associated product during the exponential and stationary phases, according to the Luedeking–Piret equation. The values of the best-fit model parameters (µ_max_, *C*_Xm_, *m*_S_, α, and β) were illustrated in Table 1.

From the estimated model parameters, as shown in Table 1, it is clear that the BBF002 culture showed the highest maximum biomass concentration (*C*_Xm_) (19.891 g/L) and maximum specific growth rate (µ_max_) (1.617 d^−1^) when compared with the native and BBF001 strains, confirming that such cultivation condition was optimal for cell growth. However, because of the trade-off phenotypes resulting from adaptive evolution, in addition to glucose consumption for biomass production, the BBF001 and BBF002 strains consumed glucose for biomass maintenance at higher rates than the native strain, as evident by the *m*_S_ values. Moreover, differences in the model parameter values related to DHA production were observed among the native, BBF001, and BBF002 strains. DHA production in the native and adaptive strains during the growth phase was higher than in the stationary phase (α > β). Therefore, DHA was the mixed-growth associated product in all strains. This result was in agreement with that of a previous study [39], wherein the same models were proposed for describing growth, glucose consumption, and DHA production of *Schizochytrium limacinum* OUC88. Cell growth fitted well with the logistic model generated, with µ_max_ and *C*_Xm_ at 0.08 h^−1^ and 24.83 g/L, respectively. Glucose was the substrate, which was consumed to produce biomass, intracellular metabolites including DHA, and biomass maintenance. The values of glucose uptake model parameters were *Y*_X/S_ of 0.46 g/g and *m*_S_ of 0.005 g/g h. The Luedeking–Piret equation used for DHA production obtained α and β values of 0.139 g/g and 0.001 g/g h, respectively.

## 4. Conclusions

In the present study, after the adaptive evaluation via a long-term serial transfer procedure using acidic and stepwise increases in temperature stress inducers, the ALE-T34 strain was chosen for further adaptive evolution at 34 °C for 30 cycles to achieve improved performance with the stable phenotypic trait, and was named *A. limacinum* BBF002. This adaptive strain displayed superior growth capabilities under acidic and high-temperature conditions over the native and acid-tolerant (BBF001) strains, which exhibited high biomass and DHA titers when grown at pH 4.5 and 30 °C. The comparative kinetic modeling of growth and DHA production in the BBF002, native, and BBF001 strains showed that the generated kinetic models with optimal model parameters fitted well with the experimental data for the three strains. Phenotypic traits, including biomass, lipids, DHA, and other intracellular metabolite products, as well as biomass maintenance, could be adequately described by a logistic model using glucose as a carbon substrate. According to the Luedeking–Piret equation, DHA was a mixed-growth product.

## Figures and Tables

**Table 1:** The model parameters estimated by fitting the models to the experimental data obtained from the native, BBF001, and BBF002 cultures at pH 4.5 and 30 °C.

Kinetic Models		Parameters	Strains		
			Native	BBF001	BBF002
Cell growth	$\frac{dC_{X}}{\mathrm{dt}}=µC_{X}$ $;\;µ\;=\mu_{\max}\left(1-\frac{C_{X}}{C_{\mathrm{Xm}}}\right)$	µ_max_ (d^−1^)	1.568	1.585	1.617
		*C*_Xm_ (g/L)	12.934	17.866	19.891
Glucose consumption	$\frac{dC_{S}}{\mathrm{dt}}=-\left[\left(\frac{1}{Y_{X/S}}\right)\left(\frac{dC_{X}}{\mathrm{dt}}\right)+\;m_{S}C_{X}\right]$	*Y*_X/S_ (g/g)	0.45	0.45	0.45
		*m*_S_ (g/g d)	0.005	0.024	0.033
DHA production	$\frac{dC_{P}}{\mathrm{dt}}=\;\left(\alpha \mu +\beta \right)C_{X}$	α (g/g)	0.100	0.033	0.055
		β (g/g d)	0.001	0.029	0.020

## Data Availability

The original contributions presented in this study are included in the article. Further inquiries can be directed to the corresponding author.
